# It’s Been a Hard Day’s Night and I’ve Been Working Like a Dog: Workaholism and Work Engagement in the JD-R Model

**DOI:** 10.3389/fpsyg.2019.01444

**Published:** 2019-06-21

**Authors:** Benedicte Langseth-Eide

**Affiliations:** Department of Psychology, UiT The Arctic University of Norway, Tromsø, Norway

**Keywords:** JD-R model, workaholism, work engagement, working hard, work-related health, employee well-being, KIWEST, ARK

## Abstract

The study investigates if the job-demands resources (JD-R) model could be improved by including workaholism in its health impairment process. Salient predictors and antecedents of workaholism and work engagement are identified in a sample of 12170 employees at Norwegian universities and university colleges. Structural equation modeling suggested that job demands and job resources relate to workaholism and work engagement, respectively. The results also revealed that both workaholics and work-engaged employees put in more hours at work than was expected of them. We found that workaholism was negatively related to work-related health, whereas work engagement was positively related to work-related health. These findings support the notion of workaholism and work engagement as two different forms of working hard. Finally, we tested the buffer hypothesis that job resources would moderate the effect of job demands on workaholism. The moderations were in the expected direction, but effect sizes were weaker than those typically reported in previous investigations. In conclusion, the present study supports the expansion of including workaholism in the JD-R model.

## Introduction

The unprecedented advancements in digitalization, automatization, robotization, and globalization over the past decades have impacted every line of businesses and shortened the life cycle of job content. Hence, employees need to learn and develop new skills faster than ever before. As a consequence, organizations seem to increasingly push their employees to work harder and longer (Fry and Cohen, 2009). In the pursuit of increased employee contributions, it is crucial that organizations create working conditions that enable employees to work hard and be well (Cohen and Black, 2013).

In the well-established job-demands resources (JD-R) model (Bakker and Demerouti, 2007) working conditions are positioned as predictors of well-being and ill-being at work. Recent research shows that the JD-R model could, in addition to burnout, also embrace workaholism in its account of the health impairment process (e.g., Molino et al., 2015). However, even though proposals have been made in favor of expanding the JD-R model, further investigations are needed to validate this expansion, particularly with regard to the antecedents and consequences of workaholism. Hence, in the present study, we aim to contribute to the literature on this emerging topic by identifying salient predictors of workaholism and work engagement and their relationship with overtime work and work-related health within the framework of the JD-R model.

### The JD-R Model

The main assumption in the JD-R model is that different working conditions (i.e., job demands and job resources) have a negative or positive effect on employee well-being and organizational outcomes. These effects are believed to operate via two different psychological processes. First, job demands—which are “physical, psychological, social, or organizational aspects of the job that require sustained physical and/or psychological (i.e., cognitive or emotional) effort”—lead to burnout, employee ill-being and negative organizational outcomes through the health impairment process (Schaufeli and Bakker, 2004, p. 296). Job demands may be quantitative (e.g., workload) or qualitative (e.g., emotional demands). Second, job resources—which are the “physical, psychological, social, or organizational aspects of the job that (1) reduce job demands and the associated physiological and psychological costs; (2) are functional in achieving work goals; and/or (3) stimulate personal growth, learning and development”—lead to work engagement, employee well-being and positive organizational outcomes through the motivational process (Schaufeli and Bakker, 2004, p. 296). As such, job resources may be both extrinsically motivating by providing tools or concrete information for goal achievement and intrinsically motivating by facilitating learning, growth, and development (Bakker, 2009). Previous research has revealed that a work environment with high levels of job demands and limited job resources has the highest risk of job strain (Bakker et al., 2014). In addition, the buffer hypothesis of the JD-R model states that job resources may mitigate the negative impact of job demands on employee well-being (Xanthopoulou et al., 2007).

### Working Hard

Although heavy work investment has long been a topic of interest in the scientific literature (e.g., Oates, 1971; Machlowitz, 1980; Schaufeli et al., 2006), there are vastly diverging ideas of the value and consequences of working hard. Previous research has established inconsistent associations between working hard and individual and organizational outcomes, which may be due to the notion that heavy work investment has been assessed differently (Burke and Cooper, 2008). Scholars have distinguished between two types of working hard, namely, work engagement and workaholism, which may be two constructs that can contribute to achieving construct specificity. Work engagement is typically described as a positive form of working hard, while workaholism historically has been described as both a positive and a negative form of working hard (e.g., Schaufeli et al., 2008).

#### Workaholism

Oates (1971) coined the term workaholism and defined it as “addiction to work, the compulsion or the uncontrollable need to work incessantly” (p. 1), and he argued that workaholism has a negative impact on health, happiness and social relationships. Since Oates (1971), the definitions, opinions, observations, and conclusions regarding workaholism have differed in the scientific literature. Hitherto, there is still little consensus about the conceptualization and definition of the construct other than its core feature of heavy work investment (Spence and Robbins, 1992; Harpaz and Snir, 2003).

Some authors have viewed workaholism primarily as a positive quality or behavior that involves high work motivation (Korn et al., 1987; Sprankle and Ebel, 1987). Others have included both positive and negative aspects in their conceptualization of workaholism. Spence and Robbins (1992) proposed a workaholic triad that contained three concepts of workaholism, namely, work involvement, feeling driven to work because of inner pressures and enjoyment of work. Based on this, the authors distinguished among three types of workaholics: work addicts (high on involvement and feeling driven, low on work enjoyment), work enthusiasts (high on work involvement and work enjoyment, low on feeling driven) and enthusiastic addicts (high on all three concepts). In contrast, other scholars have excluded positive components from their conceptualization of workaholism and view it as a primarily negative construct (e.g., Schaufeli et al., 2009; Andreassen et al., 2012). Hence, when assessed empirically, workaholism may or may not include both negative and positive components, which might explain the discrepancies in the findings and the conceptual confusion that still exists about the nature of workaholism. Porter (1996) argued that the lack of a definition hinders the effort to research workaholism. She suggests that to overcome this problem, investigators should return to the starting point and consider workaholism as an addiction that is excessive and has harmful consequences, which would make it possible to find constructive responses. In our work, we adopt the view of Schaufeli and colleagues who defined workaholism as “the tendency to work excessively hard and being obsessed with work, which manifests itself in working compulsively” (Schaufeli et al., 2009, p. 3). This definition includes both a behavioral component (excessive work) and a cognitive component (being obsessed with work). Hence, the definition includes the core constructs that have been identified across various definitions, namely, working excessively and being obsessed with work.

Some authors argue that workaholism is linked to stable individual characteristic and claim that personality traits and values play a major role in stimulating obsession with work (e.g., McMillan and O’Driscoll, 2006; Liang and Chu, 2009). Others view workaholism as a behavioral addiction and have argued that working conditions play a role in stimulating it (e.g., Fry and Cohen, 2009; Molino et al., 2015). And some suggests that a combination of individual characteristics and working conditions may generate workaholism (Mazzetti et al., 2014). Hence, in the literature it is acknowledged that workaholism may be associated with individual characteristics as well as environmental factors. In our investigation of the role of workaholism in the JD-R model we examine the relationship between job demands (i.e., working conditions), workaholism and its consequences.

#### Work Engagement

Work engagement is defined as a “positive, fulfilling, work-related state of mind that is characterized by vigor, dedication and absorption” (Schaufeli et al., 2002, p. 74). Vigor refers to mental resilience and high levels of energy while working, persistence even in difficult phases and willingness to invest effort into one’s work. Dedication is characterized by enthusiasm about and involvement in one’s work. Absorption refers to fully concentrating on and being happily engrossed in work such that time passes quickly and one has difficulties detaching (Bakker et al., 2008). May et al. (2004) operationalize work engagement in a similar three-dimensional concept (physical, emotional, and cognitive components). Although the labels differ slightly, the physical component (e.g., “I exert a lot of energy performing my job”), emotional component (e.g., “My own feelings are affected by how well I perform my job”) and cognitive component (e.g., “I am rarely distanced when performing my job”) correspond to Schaufeli et al. (2002) emphasis on vigor, dedication and absorption. According to Harter et al. (2002), work engagement assumes both cognitive and emotional antecedents to improve work-related affective and performance outcomes. These authors conceptualize work engagement as individuals’ involvement in, satisfaction with and enthusiasm for work, which closely resembles other authors’ definitions and operationalizations of the construct. Thus, for work engagement, there seems to be general agreement among scholars.

### The Role of Job Demands for Workaholism

An abundance of research has revealed a positive relationship between job demands and burnout (e.g., Demerouti et al., 2001a; Schaufeli and Bakker, 2004; Hakanen et al., 2008). Although there have been far fewer studies on the relationship between job demands and workaholism, the results point in a similar direction (e.g., Molino et al., 2015; Mazzetti et al., 2016). Several studies have revealed that work-related factors can generate or boost workaholism, such as leaders who set the example of working hard, rewards for working hard (Van Wijhe et al., 2010), work load and time pressure (Schaufeli et al., 2008) as well as career barriers, career commitment, and career insecurity (Spurk et al., 2016).

In the present study, our hypothesis on the relationship between job demands and workaholism will be tested by combining three job demands, namely, illegitimate tasks, role conflicts, and interpersonal conflicts.

Illegitimate tasks are tasks that are perceived by the employee to exceed his or her responsibilities and that break the norm of what can be reasonably expected from a person (Semmer et al., 2010). Illegitimacy may result from being asked to do a task that typically would be carried out by others or from being asked to do a task perceived as irrelevant or unnecessary. Previous research has revealed that illegitimate tasks cause employee strain, such as anger, indignation, and a threat to the self (Semmer et al., 2015). In addition, the perceived illegitimacy of one’s workload may contribute to strain that exceeds the workload levels alone (Ford and Jin, 2015). Previous studies have shown that workaholics may perceive job tasks as more frustrating and even as a punishment given to them (Clark et al., 2014a) and that workaholism may develop in response to low self-worth (Mudrack, 2006).

Interpersonal conflicts refer to negative interactions with others in the workplace and have been associated with employees’ perceived divergence of interests or goals (De Dreu and Weingart, 2003) and occur in work environments where employees compete for resources (Kippist and Fitzgerald, 2009; Jaramillo et al., 2011). Previous research has revealed that a work culture that encourages peer competition (Liang and Chu, 2009) and “winner-takes-all” reward systems (Ng et al., 2007) are positively associated with workaholism.

Role conflicts occur when employees receive inconsistent or conflicting information concerning their job tasks. Such information could come from multiple individuals or a single person within the organization (Nixon et al., 2011). Role conflict involves a sense that things at work should be done “properly” and in a different manner. Previous research has revealed that workaholics may have a desire to do things “differently” and that they often believe that the ideal person to be in charge is one self and may even actively intrude in the work of others in order to fulfill this desire (Mudrack and Naughton, 2001).

Taken together, this leads us to propose the following hypothesis:

H1: Job demands (illegitimate tasks, interpersonal conflicts and role conflicts) are positively related to workaholism.

### The Role of Job Resources for Work Engagement

Previous studies have consistently shown that job resources, such as support from coworkers and supervisors, job control, autonomy, performance feedback, skill variety and learning opportunities, are positively associated with work engagement (e.g., Schaufeli and Salanova, 2008; Albrecht, 2011; Bakker, 2011). Moreover, a longitudinal study revealed a reciprocal relationship between job resources and work engagement in which engaged employees are successful in mobilizing their own job resources over time (Llorens et al., 2007).

The relationships between various job resources and work engagement are in accordance with the job characteristics theory (Hackman and Oldham, 1980). This theory proposes that particular core job characteristics, such as skill variety, task identity, task significance, autonomy and feedback, generate positive work-related outcomes, of which intrinsic motivation resembles the concept of work engagement. In a similar vein, self-determination theory (Ryan and Deci, 2000) posits that job resources fulfill the basic human needs of autonomy, competence, and relatedness. If these needs are satisfied, they will lead to increased intrinsic motivation and optimal functioning. Furthermore, these needs are essential for psychological health and well-being.

In the present study, our hypothesis on the relationship between job resources and work engagement will be tested by combining three job resources, namely, independence in task completion, social community at work and goal clarity.

Independence in task completion involves a sense of knowing what the job tasks entails and when the tasks can be considered completed. As such, it provides employees with control over their own work tasks (Näswall et al., 2010). Control over ones work has been recognized as an important resource among most influential models in the literature on occupational stress and health (e.g., job demands-control model, Karasek, 1979; self-determination theory, Ryan and Deci, 2000; and the JD-R model, Bakker and Demerouti, 2007) that fosters motivation and promotes work engagement.

Social community at work may provide employees with social support, by feeling cared for and appreciated and by having access to direct or indirect help, which may provide additional resources provided by colleagues and supervisors (Kossek et al., 2011; Taipale et al., 2011). Numerous studies have revealed that social community may start a motivational process that generates work engagement (e.g., Bakker and Demerouti, 2008).

Goal clarity provides the employee with a clear purpose and goal for his or her work (Arnetz, 2005; Näswall et al., 2010). Several studies have revealed that goal clarity promotes a sense of meaningful work and increases work motivation and engagement (e.g., Wright, 2001; Hansson and Anderzén, 2009; De Vreede et al., 2013).

Taken together, this leads us to propose the following hypothesis:

H2: *Job resources (i.e., independence in task completion, social community and goal clarity) are positively related to work engagement*.

### Consequences of Working Hard

One of the most obvious characteristics of workaholics is that they spend a great deal of their time working, beyond what is required of them (Schaufeli et al., 2009; van Beek et al., 2011). Employees who report high work engagement also put in more hours at work than what is expected of them (Schaufeli et al., 2008). Several studies have shown that working long hours may have a negative impact on employees’ health and well-being (e.g., Sparks et al., 1997). Interestingly, research has also found positive relationships between working overtime and health and well-being (e.g., Beckers et al., 2004; Schaufeli et al., 2008). These seemingly contradictory findings might be explained by several factors. Several studies on extremely long working hours (i.e., working 61 h or more a week) have reported that overtime work can severely affect health (e.g., Uehata, 1991; Kawakami and Haratani, 1999; Amagasa et al., 2005). The associations between moderate overtime work and well-being are more complex and seem to depend on other factors. For example, Beckers et al. (2004) found that moderate overtime hours were related to fatigue when employees reported relatively adverse work characteristics, while non-fatigued employees reported relatively favorable work characteristics and high work motivation. Along a similar line, Van der Hulst et al. (2006) found that moderate overtime work only related to ill-being when employees reported high job demands in combination with low job autonomy. Thus, it seems that it is more than merely working long hours that account for the differences between individuals who work hard but are healthy and those who work hard and are in distress.

Work is recognized as an important health determinant (Waddell and Burton, 2006) and it is recognized that good health is fostered where people are gainfully employed (i.e., where the impact of work and the work environment are positive) (Buijs et al., 2012). Several authors have linked workaholism with detrimental consequences for the employee, such as a higher level of job stress (Spence and Robbins, 1992), conflicting relationships with colleagues (Schaufeli et al., 2006), work-home conflicts (Clark et al., 2014a) and impaired social relationships outside of work (Burke and Cooper, 2008). In addition, workaholics reports higher levels of ill-being, such as burnout (Taris et al., 2005), poor subjective well-being (Schaufeli et al., 2006) and decreased physical and mental health (Clark et al., 2014b). On the other hand, previous studies have suggested that employees who are highly engaged perform better (Salanova et al., 2005; Bakker and Bal, 2010; Christian et al., 2011), show more positive extra role behaviors such as citizenship behavior (Babcock-Roberson and Strickland, 2010), are more committed to their organization (Hakanen et al., 2008), and have increased innovativeness and lower turnover intention (Bhatnagar, 2012). Moreover, engaged workers report fewer psychosomatic complaints (Demerouti et al., 2001b) and better self-reported health (Hakanen et al., 2006) and suffer less from self-reported headaches, cardiovascular problems and stomach aches (Schaufeli and Bakker, 2004). In other words, engaged employees seem to enjoy good mental and psychosomatic health (Schaufeli and Salanova, 2008).

These assumptions can be empirically tested:

H3a: Workaholism mediates the relationship between job demands and overtime work.
H3b: Work engagement mediates the relationship between job resources and overtime work.
H4a: *Workaholism mediates the relationship between job demands and perceived work-related health*.
H4b: Work engagement mediates the relationship between job resources and perceived work-related health.

### The Buffer Hypothesis

When testing the buffer (moderation) hypothesis of the JD-R model, that job resources may reduce the impact of job demands on workaholism, we combine one job resource and one job demand and their interaction effect on the relationship between job demands and workaholism.

Some scholars have proposed that specific job resources should match the job demands in the workplace to reduce the impact of the demands, also known as the matching hypothesis (Frese, 1999; De Jonge and Dormann, 2006). The matching hypothesis claims that only cognitive resources will reduce the negative impact of cognitive demands, whereas emotional and physical resources are beneficial in reducing the strain due to emotional and physical demands, respectively. However, several studies applying the JD-R model have found that job resources can buffer the impact of largely independent job demands (i.e., they share little overlap) (e.g., Bakker et al., 2005, 2011; Xanthopoulou et al., 2007). It has been argued that it is difficult to label specific job demands and job resources into clear categories and that employees can perceive and experience the same job demands and job resources in different ways (Bakker et al., 2011). For example, it is possible that being given illegitimate tasks can be experienced as an increased work load (i.e., physical and/or cognitive) by some employees and as unfair (i.e., emotional) by others. This notion supports the role of job resources in the JD-R model, which claims that by definition, any job resource can buffer the impact of any demand on any type of outcome.

Hence, the following hypotheses can be articulated:

H5: H5: Job resources moderate the relationship between job demands and workaholism. Specifically, the relationship between job demands and workaholism will be stronger for employees who report low job resources than for employees who report high job resources, particularly under conditions of high job demands.

**FIGURE 1:**
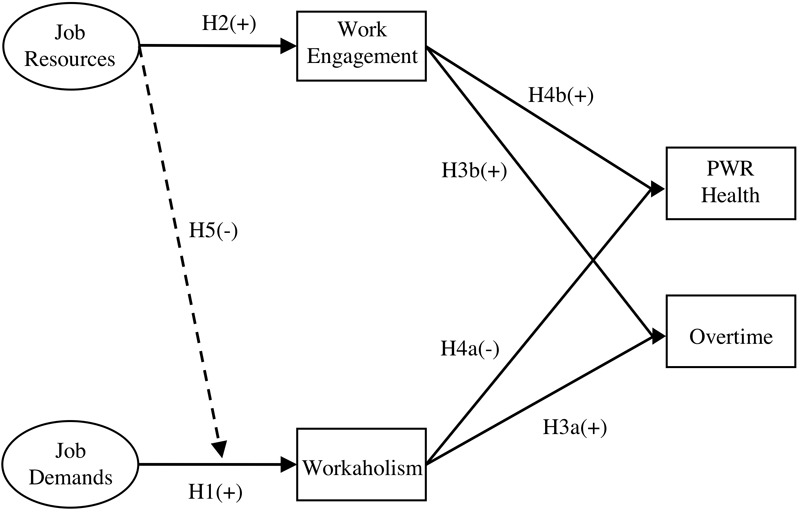
The study model including the hypotheses (H1 to H5) and direction of effect. PWR Health, perceived work-related health.

The study model is presented in Figure 1.

## Materials and Methods

### Participants and Procedure

Data were collected by ARK, a commissioned project from the Centre for Health Promotion Research at the Norwegian University of Science and Technology. The questionnaire was sent in a link via e-mail to be answered electronically. A page-long cover letter that explained the purpose of the questionnaire and ensured employee confidentiality was also included. The questionnaire could be answered over a 3-week period, in which two reminders were sent out to invitees that failed to respond.

A total of 12170 employees at Norwegian universities and university colleges participated in the study as a part of a working environment and work climate survey. Of the participants, 46.4% were men (*n* = 5642), and 53.6% were women (*n* = 6527). The ages were subdivided into five groups: < 30 years old (9.8%, *n* = 1178), 30–39 years old (23.2%, *n* = 2794), 40–49 years old (27.2%, *n* = 3271), 50–59 years old (24.3%, *n* = 2925) and > 60 years old (15.5%, *n* = 1859). Seventy-five percent of the sample consisted of permanent employees (*n* = 8979). The years of employment ranged from 0 to 50 years (*M* = 10.18, *SD* = 9.12); 45.2% of the participants were technical and administrative personnel (*n* = 5519), 37.5% were scientific and teaching staff (*n* = 4562), 11.9% were research fellows (*n* = 1452), and 5.3% were unit leaders (*n* = 637).

### Measures

All measures are drawn from the second version of the Knowledge Intensive Working Environment Survey Target (KIWEST 2.0), developed by ARK (Innstrand et al., 2015; Undebakke et al., 2015). KIWEST examines employees’ individual experiences of psychosocial working environment factors (including demands and resources). It is based on standardized and validated measures from Nordic and European research.

#### Job Demands

Job demands were measured using three scales: illegitimate tasks, interpersonal conflicts, and role conflicts.

The illegitimate tasks scale (Semmer et al., 2010) investigated the degree to which respondents experienced being given tasks outside their arena of responsibility with four items; a sample item was “I must carry out work which I feel demands more of me than is reasonable.” Responses were measured on a five-point Likert scale (1 = strongly disagree, 5 = strongly agree). Cronbach’s alpha was 0.77.

The interpersonal conflict scale (Näswall et al., 2010) measured the extent to which work was negatively affected by conflicts between employees. The scale consisted of three items, and a sample item was “In my unit, there is a great deal of tension due to prestige and conflicts.” Responses were measured on a five-point Likert scale (1 = strongly disagree, 5 = strongly agree). Cronbach’s alpha was 0.87.

The role conflict scale (Dallner, 2000) assessed the degree to which the participants perceived conflicts between their different roles with four items; a sample item was “I am often given assignments without adequate resources to complete them.” Responses were measured on a five-point Likert scale (1 = to a very small extent, 5 = to a very large extent). Cronbach’s alpha was 0.73.

#### Job Resources

Job resources were measured using three scales: task completion, social community at work, and goal clarity.

The task completion scale (Näswall et al., 2010) measured the extent to which employees could, or had to, determine when their tasks were completed. Due to statistical analyses that documented an overlap between two items, the four-item scale was reduced to three items (Innstrand et al., 2015). An example item was “I determine when my work assignments are completed.” Responses were measured on a five-point Likert scale (1 = strongly disagree, 5 = strongly agree). The Cronbach’s alpha was 0.64.

The social community at work scale was adapted from the Copenhagen Psychosocial Questionnaire II (COPSOQ II) (Pejtersen et al., 2010) and measured respondents’ degree of social community with colleagues in their own unit using three items. A sample item was “There is a good sense of fellowship between the colleagues in my unit.” Responses were measured on a five-point Likert scale (1 = strongly disagree, 5 = strongly agree). The Cronbach’s alpha was 0.83.

The goal clarity scale (Näswall et al., 2010) measured to what degree the respondents had a clear picture of the purpose of his or her own work with four items. One item was removed after statistical analyses revealed an overlap, leaving three items (Innstrand et al., 2015). A sample item was “What is expected of me at work is clearly expressed.” Responses were measured on a five-point Likert scale (1 = strongly disagree, 5 = strongly agree). The Cronbach’s alpha was 0.78.

#### Working Hard

Workaholism was measured using the Dutch Workaholism Scale (DUWAS, Schaufeli et al., 2009), which consists of 10 items. The scale covers two aspects of workaholism: working compulsively (sample item: “I often feel that there’s something inside me that drives me to work hard”) and working excessively (sample item: “It is hard for me to relax when I’m not working”). The response alternatives were 1 (almost never), 2 (sometimes), 3 (often), and 4 (always). The Cronbach’s alpha was 0.93. Schaufeli et al. (2009) suggested that working compulsively and having an exaggerated inner drive to work represent two distinct dimensions of workaholism. An exploratory factor analysis with maximum likelihood conducted on the data from the present study did not reveal a clear two-factor solution, nor did a subsequent confirmatory maximum likelihood analysis. Therefore, a one-dimensional mean score variable based on all 10 items was computed and used for the subsequent analyses.

Work engagement was measured using the nine-item version of the Utrecht Work Engagement Scale (UWES, Schaufeli and Bakker, 2003). These items covered three aspects of the work engagement concept: vigor (sample item: “At my job, I feel strong and vigorous”), dedication (sample item: “My job inspires me”) and absorption (sample item: “I get carried away when I’m working”). The response alternatives were 0 (never), 1 (a few times a year), 2 (once a month or less), 3 (a few times a month), 4 (once a week), 5 (a few times a week), and 6 (every day). The Cronbach’s alpha was 0.82. Schaufeli et al. (2002) suggested that vigor, dedication, and absorption represent three distinct dimensions of work engagement. An exploratory factor analysis with maximum likelihood conducted with data from the present study did not find a clear three-dimensional model, nor did a subsequent confirmatory maximum likelihood factor analysis. Therefore, a one-dimensional mean score variable based on all nine items was computed and used in the subsequent analyses.

#### Work Outcomes

Overtime work was assessed by asking the participants “How many hours over and beyond your agreed working hours do you normally work per week?” The response alternatives were 1 (0 h), 2 (1–5 h), 3 (6–10 h), and 4 (more than 10 h).

The perceived work-related health was measured using two items about the respondents’ experience with how their work situation impacted their health. The items were “My work has a positive influence on my health” and “My work has a negative influence on my health.” The two items correlated negatively (*r* = -0.66, *p* < 0.001). The Cronbach’s alpha was 0.80. For further statistical analyses, we reversed the item measuring negative health and computed the two items into a variable assessing the total perceived work-related health. The response alternatives ranged from 1 (to a very small extent) to 5 (to a very large extent).

### Statistical Analyses

We computed the internal consistencies (Cronbach’s α), descriptive analyses and intercorrelations among the study variables using the PASW 25.0 program.

To test the study hypotheses, we applied structural equation modeling (SEM) using the Mplus 8.0 software package (Muthén and Muthén, 1998–2017). Several goodness-of-fit criteria were considered: the root mean square of approximation (RMSEA), the comparative fit index (CFI), the Tucker-Lewis index (TLI) and the standardized root mean square residual (SRMR). RMSEA values below 0.07, SRMR values below 0.08, and CFI and TLI values greater than 0.95 indicate a good fit (Hooper et al., 2008).

**FIGURE 2:**
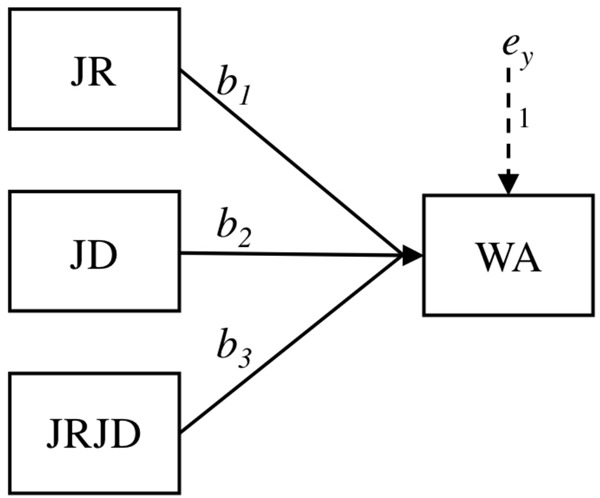
The study model for testing the interaction hypotheses. JRs, job resources; JDs, job demands; WA, workaholism.

For the moderation analyses, we applied the Hayes PROCESS macro for PAWS 25.0 (Hayes, 2017). For each hypothesized interaction effect, we tested a model that included one job demand, one job resource and their interaction, i.e., three exogenous variables. Each of the exogenous variables had only one indicator, which was the centered score of the variable. The indicator of the interaction effect was the multiplication of the interacting variables. Workaholism was included as the endogenous variable. Figure 2 represents the model used to test the interaction hypotheses.

## Results

As self-reports collected at one point in time were used in this study, Harman’s single-factor test was conducted for examining whether or not the common method bias was serious (Podsakoff et al., 2003). The results revealed that no factor explained more than 50% of the variance. This outcome suggests that common method bias did not improperly impact the model.^1^

### Descriptive Statistics

The means, standard deviations, intercorrelations, and coefficient alphas of all the included variables are presented in Table 1. As expected, workaholism correlated positively with job demands (i.e., illegitimate tasks, interpersonal conflicts, and role conflicts) and overtime work and negatively with work-related health. On the other hand, work engagement, as expected, correlated positively with job resources, overtime work and work-related health. This result is in line with hypotheses 1 and 2.

**Table 1:** Descriptive statistics, Pearson’s product-moment correlations and Cronbach’s alphas (in the diagonal) for task completion, social community, goal clarity, illegitimate tasks, interpersonal conflicts, role conflicts, work engagement, workaholism, perceived work-related health, and overtime work.

Variables	1	2	3	4	5	6	7	8	9	10
(1) Task completion	(0.64)									
(2) Social community	0.22	(0.83)								
(3) Goal clarity	0.35	0.43	(0.78)							
(4) Illegitimate tasks	-0.27	-0.38	-0.49	(0.77)						
(5) Interpersonal conflicts	-0.20	-0.62	-0.42	0.50	(0.87)					
(6) Role conflict	-0.33	-0.43	-0.55	0.70	0.55	(0.73)				
(7) Work engagement	0.23	0.35	0.36	-0.25	-0.23	-0.29	(0.82)			
(8) Workaholism	0.11	-0.17	-0.19	0.37	0.24	0.33	0.10	(0.93)		
(9) Perceived work-related health	0.21	0.41	0.39	-0.48	-0.41	-0.47	0.40	-0.37	(0.80)	
(10) Overtime work	-0.03	-0.09	-0.05	0.18	0.12	0.14	0.18	0.56	-0.16	^∗^
*N*	12023	11966	12034	11926	11958	11950	11643	11273	12034	11900
Mean	3.72	3.99	3.55	2.39	2.31	2.49	4.60	2.17	4.90	2.28
*SD*	0.64	0.76	0.77	0.76	1.02	0.72	1.04	0.56	1.41	0.90

*All correlations are significant at the 0.01 level (*p*). ^∗^ single item question.*

### Mediation Analyses

Table 2 includes the results of the SEM model estimated to test the study hypothesis. First, we conducted CFAs in which the job characteristics were loaded on one factor and two factors (i.e., job demands and job resources). The results revealed that only the model with two factors had a good fit. Hence, for the subsequent analyses the six job characteristics were modeled into two latent factors representing job demands (illegitimate tasks, interpersonal conflicts, and role conflicts) and job resources (independence in task completion, social community, and goal clarity), which were treated as exogenous variables.

**Table 2:** Fit indices of the model (*N* = 12169).

	CFI	TLI	RMSA	SRMR
CFA1 factor	0.89	0.82	0.16	0.05
CFA2 factor	0.99	0.99	0.03	0.01
M1 hypothesized	0.95	0.92	0.08*	0.06
M2 final	0.98	0.97	0.05	0.03

*^∗^The RMSEA had a *p*-value of <0.001, indicating that the hypothesized model does not have a good fit.*

**FIGURE 3:**
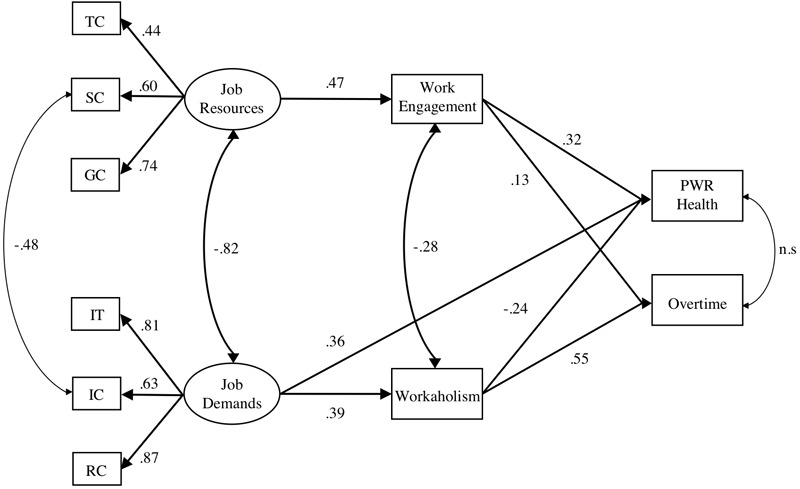
SEM model. Standardized solution. All paths are statistically significant at *p* < 0.001. TC, task completion; SC, social community; GC, goal clarity; ITs, illegitimate tasks; ICs, interpersonal conflicts; RCs, role conflicts; PWR Health, perceived work-related health.

The hypothesized mediation model (M1), in which workaholism was a full mediator between job demands and overtime work and between job demands and work-related health and work engagement was a full mediator between job resources and overtime work and between job resources and work-related health, showed a good fit to the data for two of the four criteria, namely, the CFI and SRMR. However, the TLI was slightly below the criterion value of 0.95, and the RMSEA had a *p*-value of > 0.001, indicating that the data did not fit the model. Thus, we tested a new model (M2) in which workaholism was a partial, not full, mediator between job demands and work-related health. The new model showed a good fit to the data for all four criteria. In conclusion, the results support hypotheses 1, 2a, 3a, 3b, 4a, and 4b. The final model is graphically represented in Figure 3.

### Testing Mediations

Next, the hypothesized mediating paths in the model were evaluated and are presented in Table 3. The results showed that the indirect effect of job demands on overtime work and work-related health through workaholism was statistically significant at *p* < 0.001, as was the indirect effect of job resources on overtime work and work-related health through work engagement. These results offer additional support for hypotheses 3a, 3b, 4a, and 4b.

**Table 3:** Estimates (Est.), standard errors (SE), *p*-values (p), and confidence intervals (CI) for the mediated effects (*N* = 12168).

	Est.	*SE*	*p*	CI 95%
JD → WA → PWR Health	-0.09	0.01	<0.001	[-0.10, -0.08]
JD → WA → OT	0.21	0.01	<0.001	[0.20, 0.22]
JR → WE → PWR Health	0.15	0.01	<0.001	[0.14, 0.16]
JR → WE → OT	0.06	0.01	<0.001	[0.05, 0.07]

*Parameter estimates are standardized coefficients. JDs, job demands; JRs, job resources; WA, workaholism; WE, work engagement; PWR Health, perceived work-related health; OT, overtime work.*

### Moderation Analysis

Table 4 shows the result of the nine interaction effects used to test hypothesis 5, that job resources would mitigate the positive relationship between job demands and workaholism. Eight of the nine interaction effects of job demands and job resources were statistically significant; only goal clarity did not interact significantly with interpersonal conflicts on workaholism. The positive relationship between job demands and workaholism was higher under conditions of low versus high job resources when job demands were high.

**Table 4:** Regression weights (b), confidence intervals (CI), standard errors (*SE*), *t*-values, *p*-values, and squared multiple correlations (*R*^2^) from a set of linear regression analyses with workaholism as the dependent variable, job demands as the independent variable and job resources as the moderator variable.

Predictor	*b*	CI	*SE*	*t*	*p*	*R*^2^
Constant	2.17	[2.16, 2.18]	0.005	441.02	<0.001	0.14
Task completion	-0.00	[-0.20, 0.01]	0.008	-0.26	0.797	
Illegitimate tasks	0.27	[0.26, 0.29]	0.007	39.13	<0.001	
Task completion × Illegitimate tasks	-0.045	[-0.06, -0.03]	0.009	-5.06	<0.001	
Constant	2.17	[2.16, 2.18]	0.005	428.34	<0.001	0.14
Social community	-0.02	[-0.03, -0.005]	0.007	-2.59	0.010	
Illegitimate tasks	0.27	[0.25, 0.28]	0.007	36.83	<0.001	
Social community × Illegitimate tasks	-0.021	[-0.04, -0.01]	0.008	-2.74	0.006	
Constant	2.16	[2.15, 2.17]	0.005	416.82	<0.001	0.14
Goal clarity	-0.01	[-0.02, 0.01]	0.008	-0.14	0.886	
Illegitimate tasks	0.27	[0.25, 0.28]	0.008	35.01	<0.001	
Goal clarity × Illegitimate tasks	-0.044	[-0.06, -0.03]	0.007	-6.09	<0.001	
Constant	2.17	[2.16, 2.18]	0.005	430.15	<0.001	0.06
Task completion	-0.05	[-0.06, -0.03]	0.009	-5.65	<0.001	
Interpersonal conflicts	0.13	[0.12, 0.14]	0.005	23.98	<0.001	
Task completion × Interpersonal conflicts	-0.035	[-0.05, -0.02]	0.008	-4.45	<0.001	
Constant	2.17	[2.16, 2.18]	0.006	375.80	<0.001	0.06
Social community	-0.02	[-0.04, 0.00]	0.009	-1.88	0.060	
Interpersonal conflicts	0.12	[0.11, 0.14]	0.007	18.38	<0.001	
Social community × Interpersonal conflicts	-0.013	[-0.02, -0.01]	0.006	-2.02	0.043	
Constant	2.17	[2.16, 2.18]	0.005	406.44	<0.001	0.07
Goal clarity	-0.08	[-0.09, -0.06]	0.008	-10.03	<0.001	
Interpersonal conflicts	0.11	[0.10, 0.12]	0.005	19.34	<0.001	
Goal clarity × interpersonal conflicts	-0.009	[-0.02, -0.00]	0.007	-1.39	0.165	
Constant	2.17	[2.16, 2.18]	0.005	431.70	<0.001	0.11
Task completion	0.01	[-0.01, 0.03]	0.009	1.14	0.254	
Role conflicts	0.26	[0.24, 0.27]	0.008	34.43	<0.001	
Task completion × Role conflicts	-0.048	[-0.07, -0.03]	0.100	-5.00	<0.001	
Constant	2.17	[2.16, 2.18]	0.005	414.91	<0.001	0.11
Social community	-0.02	[-0.03, -0.002]	0.008	-2.18	0.029	
Role conflicts	0.25	[0.23, 0.26]	0.008	31.63	<0.001	
Social community × Role conflicts	-0.021	[-0.04, -0.004]	0.008	-2.46	0.014	
Constant	2.16	[2.15, 2.17]	0.005	400.92	<0.001	0.11
Goal clarity	-0.001	[-0.02, 0.01]	0.008	-0.16	0.871	
Role conflicts	0.25	[0.23, 0.27]	0.009	29.52	<0.001	
Goal clarity × Role conflicts	-0.039	[-0.06, -0.02]	0.008	-4.88	<0.001	

*n = 12030–12051. *p* < 0.001 for all overall models.*

**FIGURE 4:**
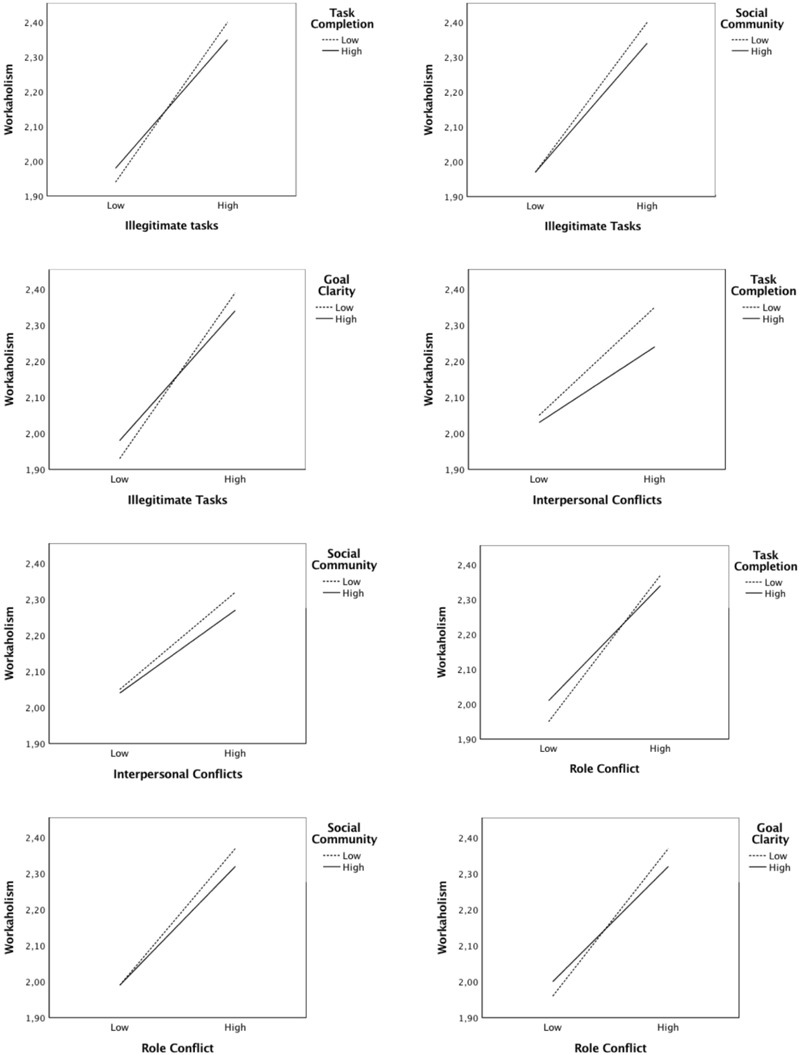
The interaction effects.

The directions of the interactions were as expected. Figure 4 shows the directions of the eight significant moderation effects.

## Discussion

The aim of the current study was to investigate whether workaholism could be included in the JD-R model. Hence, we examined antecedents and consequences of workaholism and work engagement within the framework of the JD-R model.

We assumed that different working conditions would have a negative or positive effect on employee well-being and hypothesized that job demands and job resources would be positively related to workaholism and work engagement, respectively (H1, H2). As expected, our results revealed that job demands predicted workaholism and that job resources predicted work engagement. Our findings support the main assumption of the JD-R model, namely, that different working conditions (i.e., job demands and job resources) may have a negative or positive effect on employee well-being. Our final model also supports the notion that environmental factors may play a role in generating or boosting workaholism. Thus, it is likely that a work environment that promotes workaholic behavior increases the chances of producing workaholics, while a work environment rich in resources enhances the chances of generating engaged workers.

Further, we examined the consequences of workaholism and work engagement. We hypothesized that workaholism and work engagement would mediate the relationship between job demands and overtime work (H3a) and between job resources and overtime work (H3b), respectively. In line with previous research, we found that both workaholic and engaged employees put in more hours at work than was expected of them. More specifically, the results suggest that workaholism was a stronger predictor for overtime than work engagement. We also hypothesized that workaholism would mediate the relationship between job demands and work-related health (H4a). This hypothesis was not confirmed completely, as only a partial mediation of workaholism was observed rather than the hypothesized full mediation. Our results suggest that workaholism has a negative impact on work-related health. The observed additional direct effect of job demands on work-related health is in line with literature indicating that negative working conditions have a depleting effect on employee health (e.g., Westgaard and Winkel, 2011; Rugulies, 2012). Furthermore, we hypothesized that work engagement would mediate the relationship between job resources and work-related health (H4b). Indeed, our results confirmed that work engagement had a positive impact on work-related health. These results reveal that working hard does not necessarily have detrimental consequences. If overtime work is performed by engaged employees with access to a work environment rich in resources, work can influence one’s work-related health positively. In contrast, when working extra hours is fueled by workaholic behavior by employees in adverse working conditions, work may influence work-related health negatively. Our findings also support the distinction between workaholism and work engagement as a negative and positive form of working hard, respectively.

Lastly, we tested the buffer (moderation) hypothesis of the JD-R model and hypothesized that job resources would lessen the effect of job demands on workaholism (H5). In line with studies applying the JD-R model that found that job resources can mitigate the impact of largely independent job demands (i.e., they share little overlap), we tested all nine interaction effects. Our results confirmed the hypotheses in eight of the nine combinations between job demands and job resources. Additionally, all significant effects were in the expected directions. However, the expectation that under highly stressful working conditions the risk of workaholism should be lower if sufficient job resources are available was weaker than anticipated. There might be several reasons for this result. Previous research has revealed that in their attempt to continue working, workaholics may even go as far as actively creating more work for themselves, for instance, by making their work more complicated than necessary or by refusing to delegate job tasks (Kanai and Wakabayashi, 2001; Schaufeli et al., 2009). In addition, it has been revealed that workaholics may perceive their workplace environment as being more demanding and stressful than others do (Bakker et al., 2009). Moreover, it has been reported that workaholics are inflexible, rigid, and perfectionists (Schaufeli et al., 2006). Taken together, this may imply that workaholics either cannot or do not want to use job resources, even though these resources are available to them. Furthermore, the buffer hypothesis has received an abundance of support regarding the relationship of the effects of resources and demands on burnout. However, burnout, which is a state of exhaustion and disengagement (Bakker et al., 2014), and workaholism are two different constructs. It might be that job resources are more effective to moderate the impact of job demands on burnout compared to that on workaholism.

In summary, our results suggest that different working conditions (i.e., job demands and job resources) can have a negative or positive impact on employee well-being through two different processes. Both workaholics and engaged workers put in more hours at work than what was required of them, but workaholism and job demands predicted negative work-related health, whereas work engagement predicted positive work-related health. Job resources buffered the impact of demands on workaholism in eight of the nine combinations in the expected directions, although the effect was smaller than expected. Our findings also emphasize the importance for construct specificity, i.e., that it is suitable to distinguish between a positive and a negative form of working hard (i.e., work engagement and workaholism).

Note that we use causal langue in describing and reporting the results from the mediating and moderating models. The reason is that causality is an intrinsic part of such models (e.g., Hayes, 2017). However, the causality implied by claiming that an independent variable has an effect on a mediating variable, and that both the independent and the mediating variables have causal effects on a dependent variable refers to a theoretical assumption inherent in regression models, even if the causality is not tested empirically (e.g., Davis and Weber, 1985). Despite the framing of the results in terms such as cause and effect, the results should not be interpreted as if a causal direction between these variables has been proven.

### Limitations and Future Research

Although this study has made significant contributions to the literature, some limitations need to be acknowledged. The findings come from a study with a cross-sectional design; thus, it is not possible to make causal inferences about the relations between study variables. Future studies could employ a longitudinal design to examine the causal effects of the proposed processes.

Second, all data were obtained from questionnaires, with the limitations inherent to this method. The results are also based solely on single-source data, namely, self-ratings. Future studies could add objective indicators to rule out the potential effects of common method variance. For instance, observer ratings have previously been used to study working conditions (Demerouti et al., 2001b) and could be used in future studies.

There are also limitations rooted in the measurement of subjective work-related health. First, the instrument applied measures the subjective perception of how work is influencing individual health. Other measures on health could provide better information regarding the participants general health and could provide a stronger understanding of the relationship between working hard and overall health. Second, there is some sort of norm built into questions of self-reported health. For instance, respondents may answer questions relative to similar others (e.g., my health compared with others at my age) or with respect to time (e.g., my health now compared to last year). Objective measures could overcome these methodological challenges. Finally, the study might reflect a selection bias known as “the healthy worker effect”; only those who are healthy and “survive” remain in their jobs, whereas unhealthy employees drop out. However, empirical studies suggest that problems with non-response are more severe for estimations of population means than for estimations of associations (Van Loon et al., 2003).

Additionally, the buffer hypothesis of the JD-R model was not as clear for workaholism as was previously revealed for burnout. This ambiguity should be investigated in greater detail to determine whether the relationships between job demands, job resources and workaholism are the same as those previously revealed for the relationship between job demands, job resources and burnout. Future studies could investigate whether job resources have a stronger buffer effect on burnout compared to workaholism.

## Conclusion

The present study supports the expansion of including workaholism in the health impairment process in the JD-R model. Our results offer further support for the notion that it is suitable to distinguish between workaholism and work engagement as two different types of working hard (i.e., negative and positive). Finally, our study suggests that it is possible to create working conditions which support engaged workers. This may prove to be a business advantage, providing organizations with employees who are able and willing to walk the extra mile.

## Ethics Statement

This study’s protocol was approved by the Norwegian Centre for Research Data (NSD); further approval by an Ethics Committee was not required as per applicable institutional and national guidelines and regulations. All participants gave written informed consent in accordance with the Declaration of Helsinki.

## Author Contributions

The author confirms being the sole contributor of this work and has approved it for publication.

## Conflict of Interest Statement

The author declares that the research was conducted in the absence of any commercial or financial relationships that could be construed as a potential conflict of interest.

## Footnotes

1 Detailed results are available upon request.
